# A New Way Out of the Predicament of Anaplastic Thyroid Carcinoma From Existing Data Analysis

**DOI:** 10.3389/fendo.2022.887906

**Published:** 2022-05-26

**Authors:** Yikun Zhou, Yang Zhao, Xi Ding, Jing Liang, Huayang Xu, Yuxuan Lin, Hamad Haider Khan, Bingyin Shi

**Affiliations:** ^1^Department of Endocrinology, The First Affiliated Hospital of Xi’an Jiaotong University, Shaanxi, China; ^2^Three wards of Department of Radiotherapy, Shaanxi Provincial Cancer Hospital, Shaanxi, China

**Keywords:** anaplastic thyroid carcinoma (ATC), long-term survival, radical surgery, radiotherapy, early diagnosis

## Abstract

**Background:**

Anaplastic thyroid carcinoma (ATC) is an endocrine tumor with a low incidence but a very poor prognosis. The vast majority of patients have a survival time of only three to six months, but a few survive for two years or more. In recent years, there have been major breakthroughs in targeted and immunotherapy in the field of oncology therapy. Although the preliminary study for ATC showed a promising prospect, more clinical trials are needed. It is the best approach to explore the measures that can improve survival time of ATC from the available clinical data, especially those with long survival.

**Methods:**

We report on an 82-year-old ATC patient who survived for 3 years and systematically review the clinical characteristics of 45 ATC patients with complete data from the two largest centers in northwest China. In particular, factors related to long-term survival were analyzed and summarized.

**Results:**

Three years prior, an 82-year-old woman was diagnosed with ATC by core needle biopsy following a physical examination. The thyroid tumor was resected within one month, and then the patient was treated with radiotherapy. The patient was still healthy after three years of follow-up. Analysis of prognostic factors for the 45 reviewed patients showed that those undergoing radical surgery (median overall survival (OS) = 472 days, *p* = 0.0261) and radiotherapy (median OS = 220 days, *p* = 0.0136) had better outcomes. In addition, patients younger than 65 years (median OS = 164.5 days, *p* = 0.0176) and with a lower tumor stage (IV A, median OS = 633.5 days, *p* = 0.0191) also had a better outcome.

**Conclusion:**

ATC is a highly malignant tumor, but timely early diagnosis and standardized treatment with radical surgery and radiotherapy as the core can achieve good results. Some patients can achieve long-term survival.

## Introduction

Thyroid cancer (TC) represents the two extremes of human malignant tumors. Most thyroid cancers, especially papillary thyroid cancer (PTC), which accounts for nearly 90% of all TCs, have an excellent prognosis, with a five-year survival rate of over 95% (1, 2). Anaplastic thyroid carcinoma (ATC) accounts for only 1–2% of all cases of TC, however, up to 39–50% of TC-related deaths are attributed to ATC (3–8). The unsatisfactory outcome of ATC is represented by limited improvements in survival, with reported median 1-year and 5-year overall survival (OS) rates of approximately 20% and 7%, respectively (9–12). Because of their high mortality, all ATCs are classified as stage IV by the American Joint Committee on Cancer (AJCC) (13).

In recent years, many important changes have taken place in the treatment of malignant diseases, especially targeted therapy and immunotherapy, which have improved the prognosis of many malignant tumors. Some advances have also been made in the treatment of ATC (8, 14–16). Dabrafenib plus trametinib has been approved by the Food and Drug Administration (FDA) for the treatment of BRAF^V600E^-mutated ATC (17, 18). Phase II of the Rare Oncology Agnostic Research (ROAR) study has also shown significant clinical benefits. Anti-PD-L1 antibody immunotherapy, alone or combined with a BRAF inhibitor, has also demonstrated promising results in the treatment of ATC (19, 20). However, another phase II clinical trial suggests that Lenvatinib alone may not be an effective treatment for ATC, and further investigation may be warranted (21).

An analysis of previous literature has consistently shown that a small proportion of patients with ATC will have a good prognosis, and early diagnosis and reasonable treatment can result in a longer survival, even more than 10 years (22, 23). This suggests that there is always a subset of patients who could benefit greatly from existing treatments. Given the rarity of the disease, it is difficult to conduct prospective studies and there are few experienced physicians. Analytical research on all available information, including meaningful cases, remains invaluable.

In this article, we report a case of an 82-year-old woman– *“index case”* –who had a thyroid nodule on physical examination three years prior and was soon diagnosed with ATC by fine needle aspiration cytology. Radical surgery and subsequent radiotherapy were performed without delay. After three years of follow-up, the patient was healthy and in good general condition. According to the disease itself and her age, this elderly patient should be among the patients with the worst prognosis. In the clinical analysis of 45 patients at two major medical centers in northwestern China, we further identified important factors associated with long-term survival. Under modern medical conditions, it is possible for many patients to receive an earlier diagnosis and the corresponding treatments related to long-term survival, thus obtaining a longer survival time, or even cure. For a significant proportion of patients, there is a need to move away from palliative care, which has been used in most cases in the past, toward proactive treatment and care, and strive for long-term survival for many more patients in the future.

## Methods

### Data Collection for the *“Index Case”*

Clinical data were collected after informed consent was obtained, and regular follow-up was conducted for three years.

### Collection of 45 Patients’ Demographic and Clinicopathological Data

Following approval by the corresponding institutional review boards, all 45 subjects pathologically proven to have ATC at two medical centers in northwest China between January 1, 2010, and October 1, 2020, were enrolled. After inclusion in this study the pathological results were reassessed and identified. The medical records were reviewed, including the time of diagnosis, sex, age at diagnosis, tumor pathology, TNM stage, treatment regimen, follow-up, and survival duration from diagnosis. TNM stage was determined according to the AJCC (2017) staging system based on the pathological and radiographic extent of the tumor. Tumor size was determined as the visible maximal diameter either during surgery or *via* radiological imaging [usually computed tomography (CT)].

### Treatment and Survival

Treatment strategies included surgery, chemotherapy and radiotherapy. The patient could receive one of these treatments alone or in combination with two or more multidisciplinary options. Patients came to the hospital for a follow-up visit every three months for the first year after surgery, followed by every six months for stable patients. Individual patients were followed up in different ways. The outcome index was OS, however, in this study, all patients died due to ATC, so OS was the same as disease specific survival.

Radical surgery was defined as surgical removal of all tumor tissues under the naked eye intraoperatively and no tumor tissue infiltration at the incision margin according to postoperative pathological results. Palliative surgery was defined as any surgery other than radical dissection, e.g., cytoreductive surgery or tracheotomy.

### Statistics

Survival was estimated by the Kaplan–Meier method, and any difference in survival was evaluated with the stratified log-rank test. Multivariable analysis with the Cox proportional hazards model was utilized to estimate the simultaneous effects of prognostic factors on survival. Statistical significance was defined for *p* < 0.05.

## Results

### Medical Records of the *“Index Case”* and All 5 Survivors’ Characteristics

The *“index case”* was an elderly ATC patient who was 79 years old at the time of presentation. Ultrasonography revealed a mixed echogenic nodule of thyroid on the right, 42 × 29 mm in size, with strong echogenic reflection spots, and a slightly hypoechoic nodule on the left, 11 × 9 mm in size, during a wellness check-up. The ultrasound diagnosis was nodular goiter. Fine needle aspiration cytology suggested the possibility of ATC, so core needle biopsy was performed immediately to confirm the diagnosis, followed immediately by radical thyroidectomy. During the operation, it was observed that the tumor had progressed downward to the mediastinum, and adhered to the muscle tissue. The tumor was completely removed along with the involved tissue according to visual observation. Postoperative pathological examination showed that the tumor had invaded the surrounding fibers and skeletal muscle tissues at the cutting edge, suggesting that the tumor stage should be classified as IVB. Twenty-three days after the surgery, the patient began to receive 27 rounds of radiotherapy (total 57.5 Gy), focusing on the tumor bed and possible residual lesions. The patient was followed up monthly for the first six months after discharge and then every 3 months, with no evidence of recurrence or metastasis. The patient remained tumor-free survival for more than 3 years (**Table 1** and **Supplementary Figure S1**).

**Table 1:** Survivor characteristics.

Patient ID	0	13	15	17	36
Gender	F	F	M	M	F
Age at diagnosis	79	57	72	52	61
OS (day)	1105	2647	892	795	2476
Thyroid biopsy	Yes	Yes	No	No	No
Biopsy result	ATC	ATC	/	/	/
Stage (AJCC)	IVB	IV A	IV B	IV A	IV B
Metastasis at diagnosis	Muscle	No	Parathyroid	No	Lymph node
Tumor size (cm)^a^	4	6	4	6	5
Surgery	Yes	Yes	Yes	Yes	Yes
Radical Surgery	Yes	Yes	Yes	Yes	Yes
Radiotherapy	Yes	No	Yes	No	Yes
Radiotherapy Time	27	/	33	/	23
Chemotherapy	No	No	No	No	Yes
Chemotherapy Regimen	/	/	/	/	EPI, DDP

OS, overall survival; AJCC, American Joint Committee on Cancer; EPI, epirubicin; DDP, cisplatinum.

a Tumor size using maximum diameter.

### Clinical Manifestations and Tumor Staging of All 45 Patients

A total of 45 patients with pathologically diagnosed ATC were included. The median age at diagnosis was 65 years. Twenty-eight patients (62.2%) were female, and 17 (37.8%) were male, with a female to male ratio of 1.65:1. Fifty-six percent of patients were over 65 years of age at the time of diagnosis. Most patients sought medical treatment for symptoms such as a rapidly enlarging thyroid mass (42.2%), hoarseness (40.0%), dyspnea (28.9%), dysphagia (13.3%), and choking cough after drinking water (6.7%) (**Table 2**).

**Table 2:** General characteristics, tumor stage and treatment.

Characteristics	Number of patients (n, %), N=45	
**Year**		
2010 - 2015	22	(48.89%)
2015 – 2020	23	(51.11%)
**Gender (female: male 1.65: 1)**		
Male	17	(37.78%)
Female	28	(62.22%)
**Age (46 - 84; Average 66)**		
< 65 years	20	(44.44%)
≥ 65 years	25	(55.56%)
**Earliest Symptoms**		
Rapidly enlarging thyroid mass	19	(42.22%)
Hoarseness	18	(40.00%)
Dyspnea	13	(28.89%)
Dysphagia	6	(13.33%)
Choking cough after drinking water	3	(6.67%)
**Tumor size (0.8 - 15 cm)**		
< 3cm	5	(11.11%)
3~5cm	14	(31.11%)
5~9cm	22	(48.89%)
≥ 9cm	4	(8.89%)
**Stage (AJCC, 2017)**		
IV A	4	(8.89%)
IV B	21	(46.67%)
IV C	20	(44.44%)
**Metastasis**	41	(91.11%)
* Adjacent structures*	24	(53.33%)
Muscle	9	(20.00%)
Trachea	8	(17.78%)
Esophagus	7	(15.56%)
Cervical vessels	4	(8.89%)
Cervical nerves	2	(4.44%)
Parathyroid	2	(4.44%)
* Cervical lymph nodes*	23	(51.11%)
* Distant metastases*	19	(42.22%)
Pulmonary	16	(35.56%)
Osseous	2	(4.44%)
Brain	1	(2.22%)
Liver	1	(2.22%)
**Treatment**		
Surgery only	24	(53.33%)
Radiotherapy only	1	(2.22%)
Chemotherapy only	2	(4.44%)
Multidisciplinary synthetic therapy	18	(40%)
Surgery and radiotherapy	4	(8.89%)
Surgery and Chemotherapy	5	(11.11%)
Radiotherapy and Chemotherapy	1	(2.22%)
Surgery, Radiotherapy and Chemotherapy	7	(15.56%)
No therapy	1	(2.22%)
**Surgery Method**		
Radical operation	7	(15.56%)
Palliative operation	33	(73.33%)
Tracheotomy	7	(15.56%)
Tracheotomy only	1	(2.22%)

AJCC, American Joint Committee on Cancer.

The tumor size ranged from 0.8-15 cm. The most frequent tumor stage was IV B (46.7%), followed by IV C (44.4%) and IV A (8.9%). Only 4 patients (8.9%) had no extrathyroid metastasis, while the remaining patients had local extrathyroid invasion (53.3%), lymph node metastasis (51.1%), or distant metastasis (42.2%) (**Table 2**).

### Treatment Measures Adopted

Twenty-seven patients (60.0%) were treated with monotherapy, including 24 (53.3%) with surgery alone, 2 (4.4%) with chemotherapy alone, and 1 (2.2%) with radiotherapy alone. The other patients (40.0%) received multidisciplinary treatment, including surgery combined with radiotherapy in 4 patients (8.9%), surgery combined with chemotherapy in 5 patients (11.1%), radiotherapy combined with chemotherapy in 1 patient (2.2%), and surgery combined with chemoradiotherapy in 7 patients (15.6%). One patient (2.2%) did not receive any treatment (**Table 2**).

Of the 40 patients who underwent surgical treatment, only 7 patients (15.6%) were treated with radical surgery, among which 5 patients were alive at the time of the analysis. Five patients (11.1%) underwent radical surgery and adjuvant therapy, and 2 patients (4.4%) were treated with only radical surgery. The remaining 33 (73.3%) had palliative surgery and all died, including 11 patients (24.4%) who underwent combined adjuvant therapy and 22 patients (48.9%) who underwent palliative surgery alone. (**Table 2**).

The tumors of 11 patients (24.4%) were judged by surgeons to be unable to be completely removed during or before the operation, 28 (62.2%) were judged to be feasible for radical operation, and 6 (13.3%) were not judged or explained.

### Prognostic Analysis Based on Patient Population Characteristics and Tumor Stage

**Figure 1:**
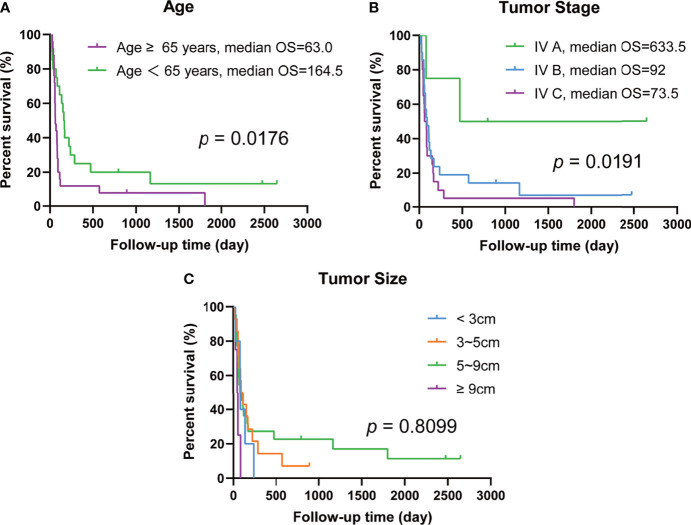
Prognostic analysis of anaplastic thyroid carcinoma patients’ baseline characteristics. **(A)** Differences in survival days between age ≥ 65 years and age <65 years. **(B)** Differences in survival days among different tumor stages. **(C)** Differences in survival days among different tumor size.

The median OS was 85 days, with a survival rate of 17.8% at 1 year and 4.4% at 5 years. The median OS according to sex was 83.5 and 110 days for males and females respectively, but this difference was not statistically significant (*p* = 0.6423). Similarly, there was no significant difference in the results of the stratified analysis based on tumor size (*p* = 0.8099, **Figure 1C**). In contrast, there was a significant difference (*p* = 0.0176, **Figure 1A**) in survival between patients younger than 65 years (median OS = 164.5 days) and those over 65 years (median OS = 63 days). In terms of tumor stage, there was a significant difference in the median OS between patients with tumors restricted to the thyroid gland (IV A, median OS = 633.5 days) and those with metastasis (IV B and IV C, median OS = 92 days and 73.5 days, respectively) (*p* = 0.0191, **Figure 1B**) (Kaplan–Meier and log-rank results shown in **Table 3** and **Figure 1**).

**Table 3:** Multivariate analysis of survival time.

Prognostic factors	Univariate analysis		
	Median OS (day)	*p* value	HR (95% CI)
**Year**		0.7386	0.90 (0.49-1.67)
2010 - 2015	84		
2015 - 2020	92		
**Gender**		0.6423	1.16 (0.62-2.16)
Female	83.5		
Male	110		
Age*^a^		0.0176	0.49 (0.26-0.92)
< 65 Y	164.5		
≥ 65 Y	63		
**Tumor size**		0.8099	
< 3cm	83		
3~5cm	102.5		
5~9cm	88.5		
≥ 9cm	50.5		
Stage* (AJCC 2017)		0.0191	
IV A	633.5		
IV B	92		
IV C	73.5		
**Treatment**			
Surgery	88.5	0.4158	0.69 (0.23-2.03)
Non-surgery	53		
Radical operation*	472	0.0261	0.37 (0.18-0.76)
Palliative operation	82		
Tracheotomy	92	0.2059	1.67 (0.62-4.46)
Non-tracheotomy	85		
Radiotherapy*	220	0.0136	0.44 (0.24-0.82)
Non-Radiotherapy	75.5		
Chemotherapy	150	0.2709	0.70 (0.38-1.30)
Non-chemotherapy	82		
**Multidisciplinary synthetic therapy**^b^			
Surgery and Radiotherapy*	220	0.0268	0.44 (0.21-0.90)
Surgery and Chemotherapy	110	0.7019	0.83 (0.33-2.09)
Surgery, Radiotherapy and Chemotherapy*	220	0.0334	0.46 (0.21-1.01)
Surgery only	81		

OS, overall survival; HR, hazard ratio; CI, confidence interval; AJCC, American Joint Committee on Cancer.

a Asterisk mark means p< 0.05.

b All patients who underwent multidisciplinary synthetic therapy were compared with patients who received surgery only.

### Prognostic Analysis of the Therapeutic Strategy

**Figure 2:**
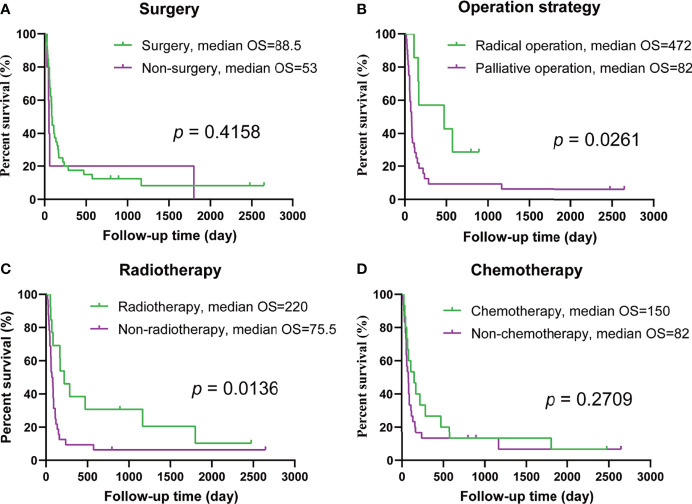
Prognosis analysis of single treatment strategy. **(A)** Overall survival (OS) in patients with and without surgery. **(B)** OS in patients undergoing radical surgery and palliative surgery. **(C)** OS in patients treated with and without radiation therapy. **(D)** OS in patients with and without chemotherapy.

The median OS of the patients’ receiving surgery was longer than that of the patients who did not undergo surgery, but there was no significant difference in prognosis between the two groups (*p* = 0.4158, **Figure 2A**). However, radical resection did improve prognosis; the median OS after radical resection was 472 days, while the median OS of the patients undergoing palliative surgery was 82 days, which was significantly worse (*p* = 0.0261, **Figure 2B**). No significant difference was found in terms of outcomes between the patients with and without tracheotomy (*p* = 0.2059). Neoadjuvant/adjuvant treatment strategies, e.g., radiotherapy, had an effect on outcomes (*p* = 0.0136, **Figure 2C**), but chemotherapy did not (*p* = 0.2709, **Figure 2D**) (Kaplan–Meier and log-rank results shown in **Table 3** and **Figure 2**).

Then, we excluded stage IVA patients, and studied the efficacy of various treatments in patients with metastasis (stages IVB&IVC). The results also showed that radical surgery and radiotherapy were effective (*p* =0.0343 and *p* =0.0008, respectively), and chemotherapy did not result in improvements in prognosis (*p* = 0.0830, **Supplementary Figure S2**).

Significant differences in outcomes were found between patients treated with surgery and radiotherapy and those treated with surgery alone (*p* = 0.0268). In contrast, no significant difference was found in patient prognosis between those receiving surgery and chemotherapy and those receiving surgery alone (*p* = 0.7019). Patients who received a combination of surgery, radiation, and chemotherapy survived significantly longer than those who received surgery alone (*p* = 0.0334). (**Table 3** and **Supplementary Figure S3**)

### Multivariable Analysis

The variables showing significant discrepancies in the univariate analysis were imported into the multivariate analysis using a Cox proportional hazards model. The protective factors correlated with to survival were confirmed to be age (< 65 years, *p* = 0.0442), operation procedure (radical surgery, *p* = 0.0213), adjuvant therapy (radiotherapy, *p* = 0.0257) and lower tumor TNM stage (*p* = 0.0289; IV A vs. IV B, *p* = 0.0197; IV A vs. IV C, *p* = 0.0407) (**Supplementary Table S1**).

## Discussion

Although ATC has long been regarded as a highly malignant and very short-lived disease, it has been reported that patients with ATC can survive for a long time, sometimes more than 10 years (22, 23). Analysis of our group of data clearly show that the core influencing factor is whether the visible tumor was radically resected, most patients who underwent radical resection had a longer survival. The longest survival time was more than seven years, which should be considered cure of the disease. Unfortunately, less than one in six patients received radical surgery in this group of patients.

The subjects with ATC recruited from the two medical centers exhibited identical prognoses. Therefore, we combined these patients into a cohort for a joint study to eliminate selection bias. The median OS was 85 days in our study, with a survival rate of 17.8% at 1 year and 4.4% at 5 years. The median OS was slightly shorter than that in previous studies, but the one-year survival was similar (24, 25). ATC was found to occur more frequently in patients over 65 years of age, and their prognosis was worse, which is consistent with previous studies (7). However, among the survivors in our study, two patients were over 65 years old, and the *“index case”* was 79 years old at the time of diagnosis, which is almost the oldest known ATC patient with tumor-free survival. As of the writing of this article, the “*index case*” is 82 years old with no metastasis or recurrence. This shows that even for patients over the age of 65 there is still a chance of better outcomes if treated properly. In this group, the incidence of ATC was higher in women than in men, but there was no significant difference in prognosis between the two sexes, which differs from the report by Rao et al., who reported that the prognosis for men is worse than for women (24). However, some studies have also shown no difference between male and female survival rates (26, 27). Additional research is therefore needed in the future.

Our data indicate that the majority of patients with IVA and IVB ATC can survive for a long time if they are treated primarily with radical surgery. Unfortunately, most of them underwent palliative surgery rather than radical surgery. It cannot be ruled out that some of them have higher surgical risks, but it is more likely that radical surgery was given up due to the subjective recognition of high malignancy after the diagnosis of ATC. To some extent, this hypothesis was confirmed in our study, where only 11 patients were judged to be truly unresectable intraoperatively or preoperatively, but only 7 of the remaining 34 patients underwent radical surgery. There are also some patients who may have the opportunity for surgery at the time of the initial visit, but at the end of the complex diagnosis process the opportunity is lost even after confirmation of the diagnosis. The *“index case”* in this group was found to have a thyroid mass during a wellness examination, and a suspicious positive diagnosis was soon established by fine needle aspiration cytology. The subsequent core needle biopsy confirmed the diagnosis, and the patient immediately underwent surgical treatment, which won a timely and expensive time window for radical treatment. As a result, this high-risk, elderly patient was able to be treated successfully in time and has been disease-free for three years.

Both these data and previous reports suggest that more than half of the cases are stage IVB or below, but most of them have a very poor prognosis because they recrive palliative care. It can be seen from the above analysis that a large proportion of these patients might be able to achieve better outcomes. In today’s highly developed society, the accessibility of medical care can lead to better outcomes for many patients, and a simple history and imaging evaluation can provide a general assessment (28). Patients who come to the hospital because of ATC usually have large thyroid masses, which can easily yield biopsy specimens for pathological examination. Although there are few surgeons with ATC surgical experience, there is no shortage of competent surgeons who have been trained due to the rapid increase in the incidence of thyroid nodules in recent decades. In addition, the development of surgical instruments also provides a certain guarantee for the radical operation of ATC. Therefore, it is now time to strive for longer survival and even cure for more ATC patients, rather than giving up, as was often the case in the past.

There was no significant relationship between tumor size and prognosis according to the analysis in this article. This is largely because ATCs grow so quickly that they outstrip the benefits of finding them sooner or later. However, early detection is a valuable opportunity for a competent medical team to perform radical treatment. The general increase in public awareness of thyroid disease and the widespread use of imaging in recent years have certainly brought new hope to ATC, which has been criticized for excessive medical examination and treatment (29, 30), as is the case for small PTCs. The tumor of the *“index case”* in this study was found during a normal physical examination, without which the patient might have lost the chance to be treated. Of course, methods for warning and discovering ATC through health examinations is the subject to be studied in the future.

It is also important to arouse public awareness of ATC, especially medical staff’s correct understanding of it. Almost all textbooks state that the survival time of ATC is only 3-6 months, which has made medical staff, patients and their families lose confidence in medical treatment (3, 8). It may be thought that short-term survival only increases the suffering of the patient and the burden on the family. Hereon, there is no denying the extremely high degree of malignancy of ATC, especially for patients with stage IVC or higher, which probably account for less than 50% of patients, and treatment may still be futile. However, the analysis in this paper shows that more than 50% of patients are stage IVB or below, and a considerable proportion of these patients could achieve a better outcome through timely and effective treatment.

In addition to the efficacy of radioactive iodine in differentiated thyroid cancer, the role of radiotherapy and chemotherapy in the treatment of TC has been controversial. However, the benefits of postoperative radiation therapy for ATC are recognized by most experts and scholars, but there is still disagreement about the effects of chemotherapy (31, 32). Therefore, comprehensive treatment (including chemotherapy) based on the individual condition of the patient with radiotherapy as the main component after surgery is another important part of improving the therapeutic effect. In this study, 24 patients only received surgery, but did not receive radiotherapy because of misunderstanding of ATC in the past. There is no doubt that radical surgery plays a central role, and in the face of such a malignant disease, any misstep can be disastrous. It should also be emphasized that treatment for ATC is also a battle for time and speed.

ATC is characterized by a higher mutation burden with increased rates of alterations in tumor suppressors (TP53), PI3K/AKT pathway genes, and the TERT promoter. TP53 mutations can be identified in up to 70% and TERT promoter mutations in approximately 40% of ATC tumors, thereby, these two mutation genes can serve as the molecular hallmarks of ATC. The BRAF^V600E^ mutation is found in 25% to 40% of ATCs. If the tumor harbors a BRAF mutation, it is PTC derived, and the prognosis is the most favorable of all types of ATC (33–35). Thus, the approach to ATC should always begin with determination of the BRAF status. Because next-generation sequencing (NGS) testing takes a long time, and the patient might lose the opportunity for treatment, BRAF gene mutations should be determined with fast immunohistochemistry (IHC) for all newly diagnosed patients with ATC. However, NGS should also be performed in parallel because approximately 60% of patients do not have a BRAF mutation (36).

Most patients with IVB disease present with advanced disease that requires radical resection, such as total laryngectomy and/or esophagectomy. Given the increased morbidity associated with radical resection, upfront surgery is avoided in most cases. In these patients, neoadjuvant treatment with a BRAF/MEK inhibitor such as Dabrafenib and Trametinib should be considered if they possess a BRAF mutation. Neoadjuvant treatment with BRAF/MEK inhibitors in patients with BRAF^V600E^-mutated ATC can significantly reduce the burden of disease and enable surgical resection. There have been reports that this approach is feasible in patients with ATC with advanced IVB disease and in selected patients whose limited distant disease responds favorably to neoadjuvant treatment. A significant reduction in disease burden can be observed within days of treatment initiation, which may improve airway and swallowing symptoms (17–20). Restaging evaluation to determine surgical candidacy can be performed after 1 to 2 months of BRAF/MEK treatment. In cases with known distant metastases, surgical resection of the primary and neck disease can be considered if distant disease has responded to neoadjuvant treatment and/or remains at a low volume. Because of the antiangiogenic properties associated with MEK inhibitors, which can impair postsurgical sound healing, MEK inhibitor are administered 5 to 7 days before surgery. BRAF inhibitors are administered the day before or on the day of surgery. Both drugs are restarted as soon as the surgical wound has healed, typically within 1 to 2 weeks after surgery. The addition of immunotherapy to BRAF/MEK is being evaluated in a neoadjuvant setting in a clinical trial (36).

One of the shortcomings of this paper is that it is a retrospective study. Although we selected cases from the past ten years, the previous cases were not included, the span of ten years is quite long, and there have been great changes in the concept of disease and treatment in that time. The second is the relatively small number of patients, especially those undergoing radical surgery. Another limitation is that no genetic testing was performed for the *“index case”*, however, it has been included in the standard testing regimen for all patients in the past year. We will carry out a prospective study to adopt a more uniform approach and administer radical surgery to as many patients as possible.

## Conclusion

In conclusion, ATC is one of the most malignant diseases. However, if patients with stage IVB disease or below are treated with radical surgery and followed-up with radiotherapy as the main component, some may have a chance for longer survival, potentially offering new hope for the management of ATC in the future. Shortening the time from the first visit to the operation and implementing radical operation are the two most important factors affecting the therapeutic effect. Targeted and immunotherapy may provide additional benefits for some patients in the future.

## Data Availability Statement

The original contributions presented in the study are included in the article/**Supplementary Material**. Further inquiries can be directed to the corresponding author.

## Ethics Statement

The studies involving human participants were reviewed and approved by Ethics Committee of The First Affiliated Hospital of Xi’an Jiaotong University. The patients/participants provided their written informed consent to participate in this study. Written informed consent was obtained from the individual(s) for the publication of any potentially identifiable images or data included in this article.

## Author Contributions

Conception and design: YZ and BS. Provision of study materials or patients: YZ, JL. Collection and assembly of data: YZ, YL and HX. Data analysis and interpretation: YZ and XD. Article writing: all authors. Final approval of article: all authors. All authors contributed to the article and approved the submitted version.

## Funding

This research was supported by National key research and development program (NO.2018YFC1311500) and the National Natural Science Foundation of China (NO.81970679).

## Conflict of Interest

The authors declare that the research was conducted in the absence of any commercial or financial relationships that could be construed as a potential conflict of interest.

## Publisher’s Note

All claims expressed in this article are solely those of the authors and do not necessarily represent those of their affiliated organizations, or those of the publisher, the editors and the reviewers. Any product that may be evaluated in this article, or claim that may be made by its manufacturer, is not guaranteed or endorsed by the publisher.
