# Supplementary material for: A New Way Out of the Predicament of Anaplastic Thyroid Carcinoma From Existing Data Analysis
Source: Front Endocrinol (Lausanne). 2022 May 26;13:887906. doi: 10.3389/fendo.2022.887906 (PMC9178175; doi:10.3389/fendo.2022.887906)
Supplement: Supplementary file 1 [file DataSheet_1.docx]

**
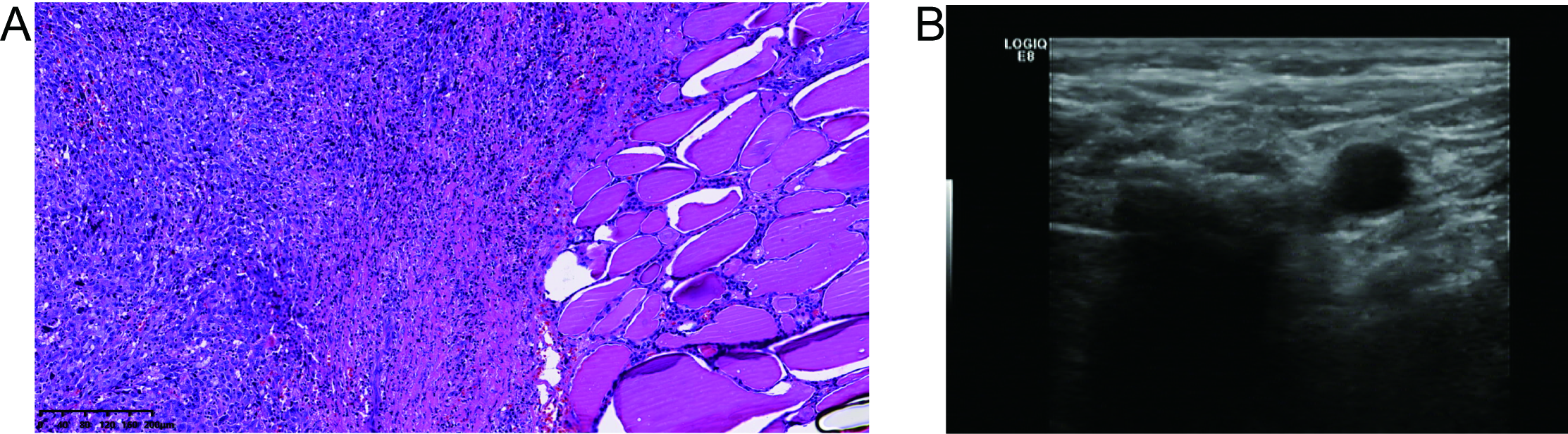
**

**SUPPLEMENTARY FIGURE. S1.** Information on “***Patient Zero”***. (**A**) Microscopic appearance of the tumor. (**B**) Results of postoperative ultrasonography (September 9, 2021). Images are presented at 20x magnification.


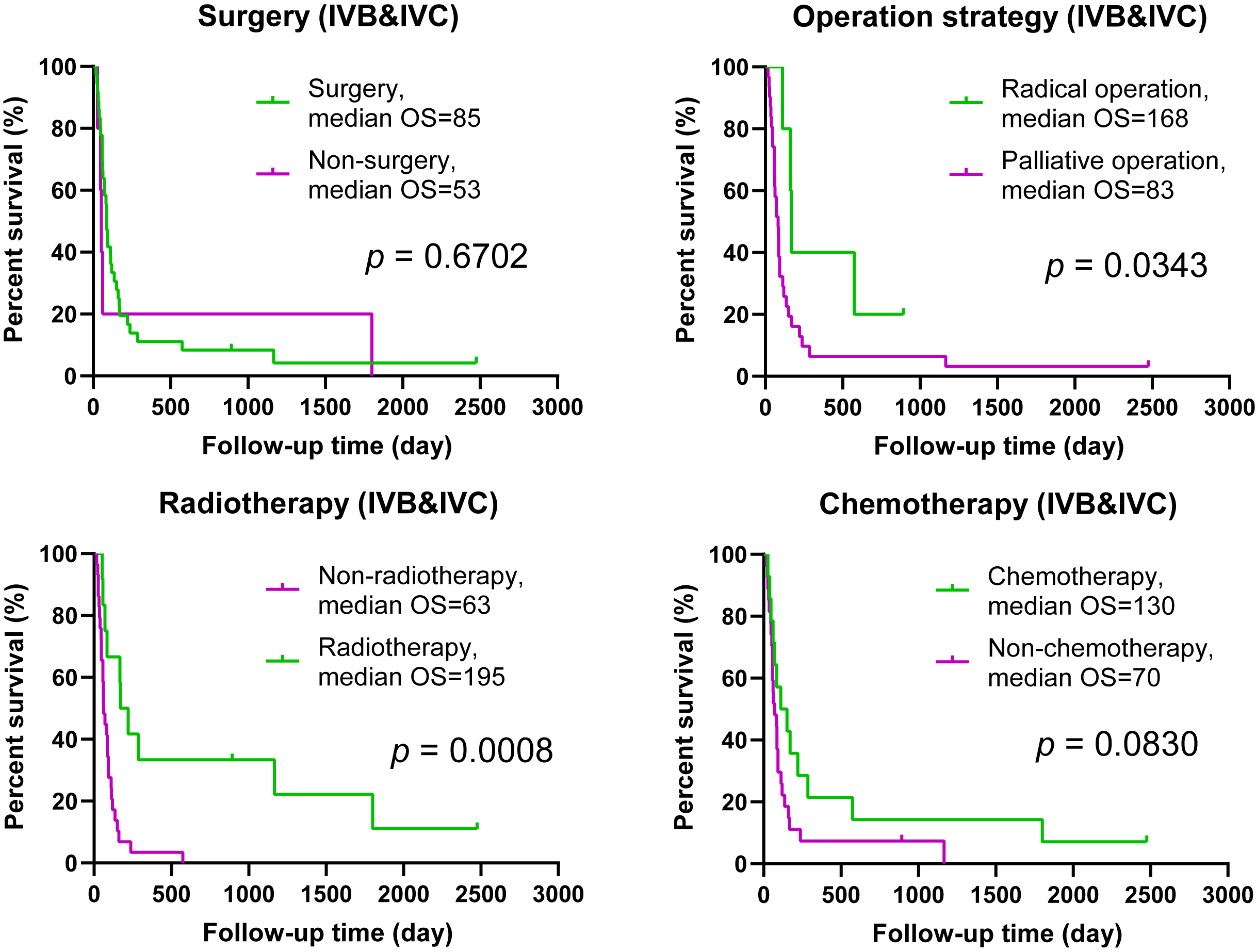


**SUPPLEMENTARY FIGURE. S2.** Prognosis analysis of single treatment strategy of patients with stage IVB&IVC. (**A**) No significant difference (*p* = 0.6702) in survival between patients who underwent surgery and those who did not undergo surgery. (**B**) Significant difference (*p* = 0.0343) in survival between patients who underwent radical operation and those who underwent palliative operation. (**C**) Significant difference (*p* = 0.0008) in survival between patients who underwent radiotherapy and those who did not undergo radiotherapy. (**D**) No significant difference (*p* = 0.0830) in survival between patients who underwent chemotherapy and those who did not undergo chemotherapy. OS, overall survival.

**
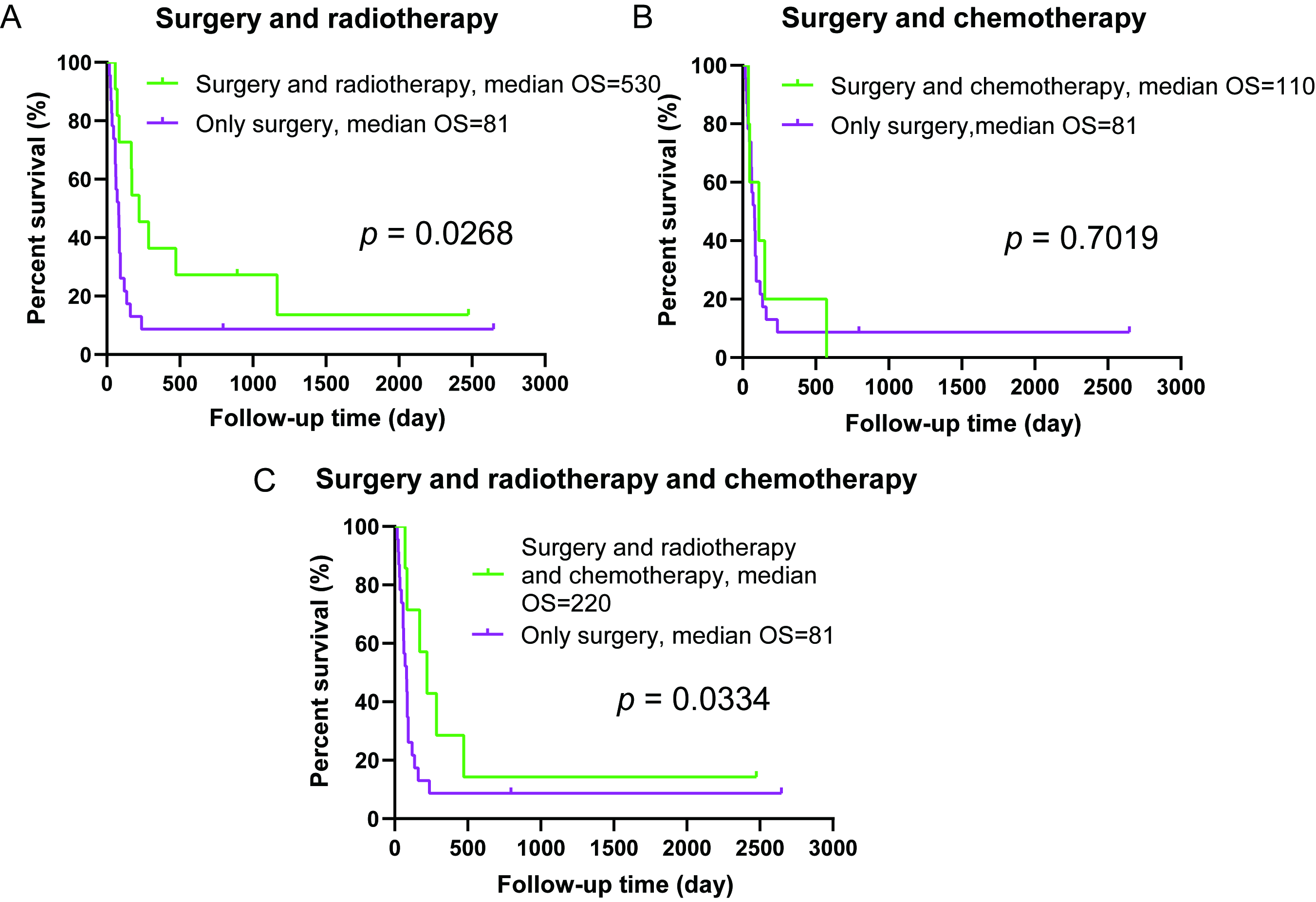
**

**SUPPLEMENTARY FIGURE. S3.** Prognosis analysis of multidisciplinary synthetic therapy strategy. (**A**) Significant difference (*p* = 0.0268) in survival between patients who underwent surgery and radiotherapy and those who underwent surgery only. (**B**) No significant difference (*p* = 0.7019) in survival between patients who underwent surgery and chemotherapy and those who underwent surgery only. (**C**) Significant difference (*p* = 0.0334) in survival between patients who underwent surgery, radiotherapy and chemotherapy and those who underwent surgery only. OS, overall survival.

**SUPPLEMENTARY TABLE S1.** Multivariable analyses of baseline and clinical characteristics for OS

| **Prognostic factors** | **Multivariate analysis** | |
| --- | --- | --- |
|  | ***p* value** | **HR (95% CI)** |
|  |  |  |
| Age ***** | 0.0442 | 0.41 (0.17-0.98) |
| Operation regimen***** | 0.0213 | 0.28 (0.10-0.83) |
| Radiotherapy ***** | 0.0257 | 0.34 (0.13-0.88) |
| Tumor Stage | 0.0289 |  |
| Stage - IV A vs IV B (AJCC 2017) ***** | 0.0197 | 0.13 (0.02-0.72) |
| Stage - IV A vs IV C (AJCC 2017) ***** | 0.0407 | 0.44 (0.20-0.97) |
|  |  |  |

Abbreviations: OS, overall survival; HR, hazard ratio; CI, confidence interval; AJCC, American Joint Committee on Cancer.

^a^ The cut-off age was 65 years; radical and palliative dissections are compared in the line of Operation regimen.

^b^ asterisk mark means *p* < 0.05.
